# Two-step staged resection of giant olfactory groove meningiomas

**DOI:** 10.1007/s00701-021-04910-3

**Published:** 2021-08-10

**Authors:** Gerhard Marquardt, Johanna Quick-Weller, Stephanie Tritt, Peter Baumgarten, Christian Senft, Volker Seifert

**Affiliations:** 1 Department of Neurosurgery, Goethe – University, Schleusenweg 2-16, 60528 Frankfurt am Main, Germany; 2 Department of Neuroradiology, Goethe – University, Frankfurt am Main, Germany

**Keywords:** Frontobasal, Olfactory groove meningioma, Peritumoural oedema, Resection, Skull base

## Abstract

**Background:**

The surgical treatment of giant olfactory groove meningiomas (OGMs) with marked perilesional brain oedema is still a surgical challenge. After tumour resection, increase of brain oedema may occur causing dramatic neurological deterioration and even death of the patient. The objective of this paper is to describe surgical features of a two-step staged resection of these tumours performed to counter increase of postoperative brain oedema.

**Methods:**

This two-step staged resection procedure was carried out in a consecutive series of 19 patients harbouring giant OGMs. As first step, a bifrontal craniectomy was performed followed by a right-sided interhemispherical approach. About 80% of the tumour mass was resected leaving behind a shell-shaped tumour remnant. In the second step, carried out after the patients’ recovery from the first surgery and decline of oedema, the remaining part of the tumour was removed completely followed by duro- and cranioplasty.

**Results:**

Ten patients recovered quickly from first surgery and the second operation was performed after a mean of 12.4 days. In eight patients, the second operation was carried out later between day 25 and 68 due to surgery-related complications, development of a trigeminal zoster, or to a persisting frontal brain oedema. Mean follow-up was 49.3 months and all but one patient had a good outcome regardless of surgery-related complications.

**Conclusions:**

Our results suggest that a two-step staged resection of giant OGMs minimizes the increase of postoperative brain oedema as far as possible and translates into lower morbidity and mortality.

## Introduction

The surgical treatment of giant olfactory groove meningiomas (OGMs; diameter >5 cm) with marked perilesional brain oedema poses special problems and is still a surgical challenge. After tumour resection, increase of brain oedema may occur causing dramatic neurological deterioration and even death of the patient. To minimize surgical morbidity and mortality, we currently favour a two-step staged resection of giant OGMs, and the purpose of this paper is to present our experience with this surgical proceeding.

## Material and methods

### Patients and methods

**Fig. 1:**
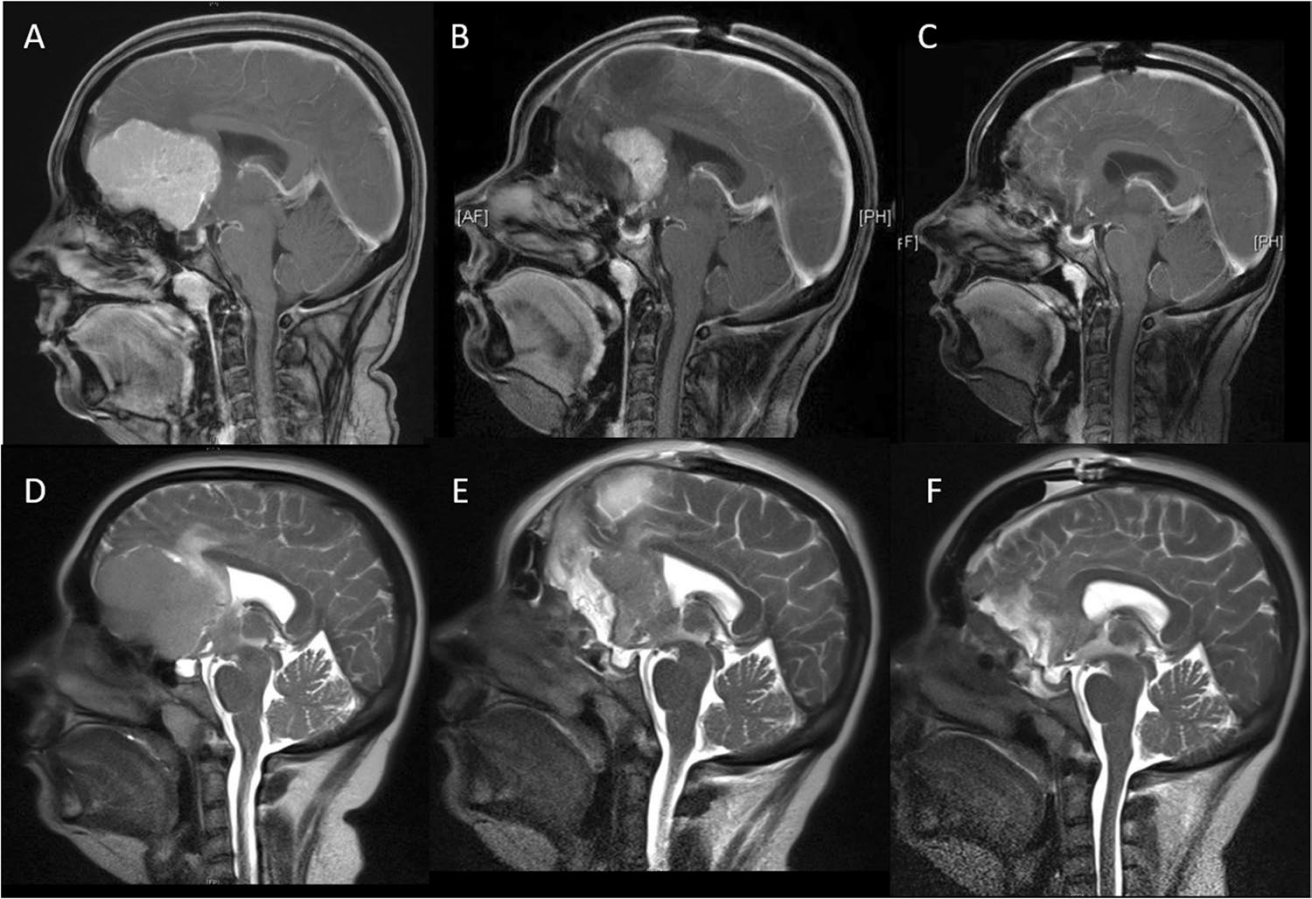
Sagittal MRI images T1-weighted gadolinium-enhanced and T2-weighted showing the perilesional oedema. **A**, **D**: Preoperative. **B**, **E**: One day after first operation showing the shell-shaped tumour remnant. **C**, **F**: One day after second operation with complete removal of the tumour

**Fig. 2:**
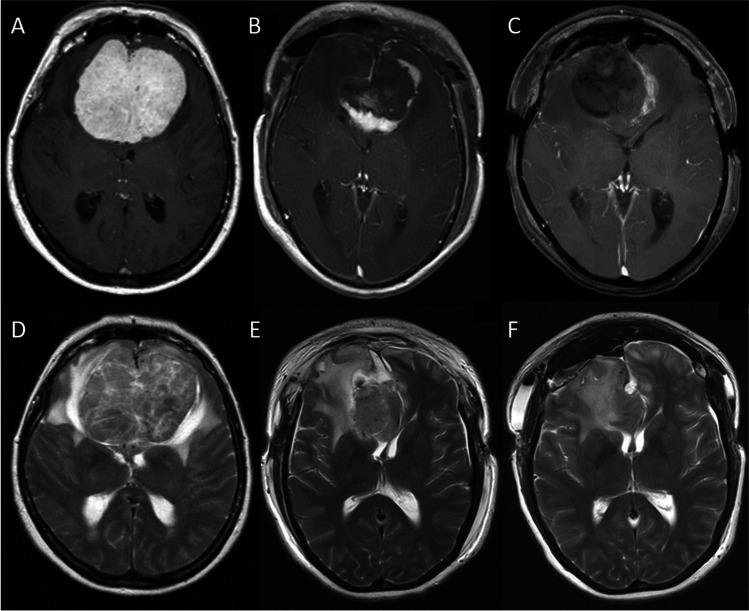
Axial MRI images. **A**, **B**, **C**, **D**, **E**, **F**: same sequences as in Fig. 1 each

The clinical and neuroradiological data of 19 consecutive patients who underwent a two-step staged resection of giant OGMs (>5 cm) at our institution between 2006 and 2018 were collected. Clinical results were compared to a consecutive series of eight patients who were operated earlier via a single-step procedure. T1-weighted gadolinium-enhanced MRI sequences were used to measure preoperative tumour dimensions (Figs. 1A and 2A).

### Surgical procedure

As first step, a bifrontal craniotomy was performed followed by a right-sided interhemispherical approach. Using microsurgical techniques and cutting the falx cerebri, about 80% of the tumour mass was resected leaving behind a shell-shaped tumour remnant (Figs. 1B and 2B). In doing so, the optic nerves were visualized as landmarks for the second operation. The lateral tumour borders and dorsal tumour aspects involving the anterior cerebral artery complex, however, were not dissected. After meticulous haemostasis and covering the olfactory groove, the wound was closed. Whereas this was realised in the first five patients with water-tight dura sutures and reinsertion of the bone flap, we changed our strategy after the fifth patient who deteriorated neurologically and eventually deceased. In the subsequent patients, a temporary craniectomy was performed, i.e. the dura was left open and the boneflap was not reinserted but stored in a deep-frozen freezer at −80°C. To avoid CSF leakage, the dura was covered with Tabotamp^®^. In the second step, carried out after the patients’ recovery from the first surgery and decline of oedema, the remaining part of the tumour was removed completely followed by duro- and cranioplasty (Figs. 1C, F and 2C, F).

## Results

There were 11 women and eight men aged 34–76 years (mean 58.4 years). The clinical presentation included anosmia, mental dysfunction and visual impairment. Mean maximal tumour diameter was 6.2 cm (range 5.1–7.6 cm) and mean tumour volume was 167.5 cm^3^ (range 95.5–292.1 cm^3^). All tumours had caused a marked perilesional brain oedema as apparent on T2-weighted MRI images (Figs. 1D and 2D).

Despite leaving behind a considerable large resection cavity after the first operation, all patients in whom a craniectomy had been carried out showed a more or less pronounced bulging of the skin flap from the second postoperative day. This was due to brain swelling and not owing to a subcutaneous accumulation of CSF (Figs. 1E and 2E). Even in the event that the frontal sinus had been opened, we did not observe rhinorrhoea since the dura had been covered with Tabotamp^®^. Only after decline of the oedema with sunken and soft skin flap when palpating, the second operation was considered in these patients. In the first four patients, in whom the bone flap had been reinserted in the course of the first operation, the decision to operate was made dependent on the clinical condition.

Ten patients recovered quickly and well from first surgery and had a soft craniectomy defect if this measure had been performed before. In these patients, the second operation, with complete tumour removal and dural as bony reconstruction, was performed between the 6^th^ and 30^th^ day (mean 12.4 days) after the first operation.

In eight patients, the second operation had to be delayed. Two of these sustained pulmonary embolism necessitating heparin treatment, and one patient developed pneumonia. Complete clinical recovery was awaited at a time before the second intervention was carried out. The same applies to two further patients who developed a trigeminal zoster. Another patient sustained a small frontal intracerebral haematoma, which was managed conservatively. Due to a persisting frontal oedema in two other patients, the execution of the second operation at this time was considered too dangerous and the patients were at first sent in a rehab centre. After decline of the oedema, the second operation was carried out 65 and 68 days respectively after the first intervention. All details are shown in Table 1.

**Table 1:** Summary of cases (two-step staged resection)

No	Age/sex	Extension of tumour in cm	Volume of tumour in cm^3^	Delay to 2^nd^ operation (days)	WHO grade	Dura left open after 1^st^ surgery	Craniectomy	Last follow-up (months)	Clinical outcome	Recurrence
1	55/f	6.7 × 6.5 × 5.1	222.1	49 *	I	No	No	19	Good	No
2	57/m	6.6 × 6.2 × 5.3	216.9	6	II	No	No	24	Good	No
3	66/f	6.4 × 4.7 × 4.1	123.3	7	I	No	No	24	Good	No
4	48/m	5.1 × 4.6 × 4.1	96.2	8	II	No	No	152	Good	1.5 × 1.5 × 1.0 cm
5	59/f	5.5 × 4.9 × 4.7	126.7	-	II	No	No	96	Poor	-
6	61/m	6.2 × 5.2 × 4.1	132.2	22	I	Yes	Yes	240	Good	No
7	64/m	7.6 × 6.2 × 6.2	292.1	12	II	Yes	Yes	5	Good	No
8	61/f	6.2 × 5.4 × 4.2	140,6	65 †	I	Yes	Yes	23	Good	No
9	76/m	5.8 × 4.8 × 4.5	125.3	30	I	Yes	Yes	12	Good	No
10	34/f	6.7 × 5.2 × 4.4	153.3	7	II	Yes	Yes	28	Good	0.8 × 0.9 × 0.4
11	59/f	7.4 × 6.6 × 4.7	229.5	11	II	Yes	Yes	56	Good	No
12	66/f	5.1 × 4.8 × 3.9	95.5	51 *	II	Yes	Yes	36	Good	No
13	60/f	6.7 × 6.5 × 6.5	283.1	6	I	Yes	Yes	49	Good	No
14	52/f	5.8 × 5.8 × 4.1	137.9	68 †	II	Yes	Yes	59	Good	No
15	57/m	6.4 × 6.1 × 4.7	183.5	25 ‡	II	Yes	Yes	48	Good	2.0 × 1.2 × 2.5 cm
16	69/f	7.1 × 7.0 × 5.2	258,4	63 §	I	Yes	Yes	3	Good	No
17	47/m	5.5 × 5.5 × 4.1	124.0	15	I	Yes	Yes	36	Good	No
18	59/m	5.5 × 4.7 × 4.5	116.3	37 ‡	II	Yes	Yes	16	Good	No
19	69/f	5.4 × 5.2 × 4.8	134.8	58 II	II	Yes	Yes	10	Good	No

*No*, consecutive number of patients; *M*, male; *F*, female; * development of Zoster; † persisting oedema; ‡ pulmonary embolism; § bronchopneumonia; II small frontal intracerebral haematoma; ¶ ventriculo-peritoneal shunt

Worth particular mention is patient No. 5. She postoperatively experienced increased brain oedema leading to sudden loss of consciousness. Despite instant decompressive craniectomy, she did not regain consciousness and eventually deceased. Accordingly, surgical complications were encountered in five of our patients. Thus, the rate of surgery-related complications was 26.4% and overall mortality was 5.3%. The clinical course of this very patient was the reason to alter our surgical strategy in the subsequent patients as described above. None of the following patients in whom a craniectomy was performed died. Thus, in this subgroup of patients, mortality was 0%.

Mean follow-up was 49.3 months (range 3–240 months). All but one patient (No. 5) had a good outcome regardless of surgery-related complications as mental dysfunction improved. This applies as well to the patient who had sustained a small intracerebral hematoma and had developed shunt-dependent hydrocephalus. Recurrence rate was 15.8%.

These clinical results were far more favourable than those obtained earlier in a consecutive series of eight patients who underwent a single-step resection of giant OGMs at our institution between 2000 and 2006. This historical control group consisted of five women and three men aged 43–73 years (mean 54.9 years). Mean maximal tumour diameter was 6.1 cm (range 5.4–7.1 cm) and mean tumour volume was 199.5 cm^3^ (range 137.7–313.9). All tumours had caused a marked perilesional brain oedema, too. Mean follow-up was 65.1 months (range 2–201 months) and recurrence rate was 14.3%. Four patients had an uneventful postoperative course and a good outcome as mental dysfunction improved. After an initially inconspicuous postoperative course, four other patients experienced a sudden coma with anisocoria on day 2, 4, 8, and 11, respectively. CT revealed an increase of the oedema each and despite immediate decompressive craniectomy the further clinical course was unfavourable in all patients without exception. Three patients survived but remained tetraparetic and complete dependent on care. Reinsertion of the bone flap was carried out approximately after three months each. One patient with multiple infarctions (cortical, thalamic, mesencephalic) deceased after termination of therapy in consultation with the relatives. Thus, overall mortality was 14.3%. All details are listed in Table 2.

**Table 2:** Summary of cases (one-step resection, historical group)

No	Age/sex	Extension of tumour in cm	Volume of tumour in cm^3^	WHO grade	Last Follow-up (months)	Clinical outcome	Recurrence
1	51/f	5.6 × 5.3 × 4.8	142.5	I	12	Good	No
2	63/f	5.4 × 5.1 × 5.0	137.7	II	6	Poor *	No
3	50/f	5.9 × 5.5 × 4.6	149.3	I	60	Good	No
4	56f	6.0 × 5.5 × 5.3	174.9	I	22	Good	No
5	45/f	6.4 × 6.2 × 5.7	226.2	I	3	Poor †	No
6	43/m	7.0 × 6.9 × 6.5	313.9	II	201	Poor ‡	2.6 × 2.3 × 2.2
7	58/m	5.5 × 5.2 × 5.0	143.0	I	152	Good	No
8	73/m	7.1 × 7.0 × 6.2	308.1	I	-	Poor §	-

*No*, consecutive number of patients; *M*, male; *F*, female; *FU*: follow-up; *VP*, ventriculo-peritoneal shunt; *po*: postoperative. * 2^nd^ po day sudden coma, anisocoria. CT: increase of oedema. Secondary craniectomy. 3 months later VP. Last FU: tetraparetic, complete dependent on care. † 8^th^ po day sudden coma, anisocoria. CT: increase of oedema. Secondary craniectomy. Po CT: bilateral infarction in areas of posterior and anterior cerebral artery. 1-month later VP. Last FU: tetraparetic, cortical blindness, complete dependent on care. ‡ 4^th^ po day sudden coma, anisocoria. CT: increase of oedema. Secondary craniectomy. 1-month later VP. Last FU: tetraparetic, dependent on care. § 11 days po sudden coma, anisocoria. CT: increase of oedema. Secondary craniectomy. Po CT: multiple infarctions (cortical, thalamic, mesencephalic). In consultation with the relatives termination of therapy. Deceased

## Discussion

Since OGMs are slow-growing lesions that develop relatively distant from the optic nerve and vital neurovascular structures [4], they may reach a very large size before producing symptoms [4, 5, 8]. These symptoms comprise mental dysfunction and visual impairment that are perceived either by the patient itself and/or by his relatives. Anosmia however, which may be considered as initial symptom and which is on determined query present for years in all patients harbouring giant OGMs, is most often not noticed by the patient.

Several approaches have been proposed for the surgical treatment of OGMs. Of the options available — standard bifrontal, extended bifrontal, unilateral subfrontal, and pterional — each has its advantages and disadvantages regarding ease, safety, avoidance of complications, and extent of exposure as Chi et al. [4] point out. For a long time the bifrontal approach was considered as the standard procedure for removal of large OGMs, whereas the unilateral subfrontal and pterional approach was used for smaller tumours [1, 4, 5, 11, 14]. By now, however, there is a vast experience of removal of giant OGMs through a pterional approach. Schaller et al., reporting their experience with the pterional approach for OGMs measuring between 3.5 and 6 cm in diameter, emphasize that total tumour resection was obtained in all but one of their 28 patients [13]. They state that the pterional approach is superior to others for these lesions since it provides early exposure of the neurovascular complex, preservation of the frontal venous drainage, and avoidance of postoperative cerebrospinal fluid fistulae. Likewise, Tomasello et al. point out that the pterional approach minimizes morbidity and mortality through an early neurovascular control and by limiting parenchymal damage [16]. 17 of their 18 patients (94.4%) with giant OGMs and treated via a pterional craniotomy had a good outcome. Similar good results with a pterional-transsylvian approach are reported by other authors [6, 17], too. El-Bahy, on the other hand, comes to a slightly different opinion [7]. With regard to tumour size he grouped his 18 patients into A (tumour size less than 4 cm in diameter) and B (tumour size more than 4 cm in diameter). In one patient belonging to group B, he encountered a marked swelling of the right frontal lobe after dural opening, which necessitated partial right frontal pole resection. Based on his experience he concludes that the frontolateral approach is a minimally invasive approach to OGMs less than 4 cm in diameter and to tumours more than 4 cm in diameter without encasement of the anterior cerebral artery complex. He points out, however, that a tumour size more than 4 cm in diameter and encasement of the anterior cerebral artery complex are limiting factors for the frontolateral approach if radical tumour removal is considered.

Chi et al. emphasize that the choice of the surgical approach is not only dependent on the size of the tumour but also on the involvement of neurovascular structures and the preference and comfort level of the surgeon [4]. Currently, we perform a frontolateral approach for smaller tumours with good results but prefer the bifrontal exposure for giant OGMs.

The considerable mass effect of giant OMGs may be increased by the perilesional oedema. Reviewing MRI scans of patients with OGMs for brain oedema, Chi et al. grouped the T2 abnormalities into four categories: A, no oedema; B, oedema restricted to the gyrus rectus (mild); C, oedema beyond the gyrus rectus (moderate); D, extensive bifrontal oedema (severe) [4].

Even though the exact pathogenesis of peritumoural brain oedema in meningiomas is still unknown [3], preoperative brain oedema is likely to be related to tumour size, with increasing oedema seen with larger tumours [4]. In the series of Nakamura et al. [11], the mean diameter of OMGs was 4.46 cm (range 1.4–10 cm), and they observed preoperative peritumoural oedema on CT or MRI scans in 48 of their 82 patients (58.6%). In our series the tumour diameters ranged from 5.1 to 7.6 cm (mean 6.2 cm), and a severe perilesional oedema according to the classification of Chi et al. [4] was present in all patients. It is important to point out that there is a close correlation between the extent of the oedema and postoperative complications [18] and that a pronounced oedema constitutes an increased risk of perioperative mortality and morbidity [9]. Moreover, the oedema is likely to increase owing to surgical manipulations. While Asgari et al. [2] reported postoperative deterioration due to an increasing oedema in only 3.5% of their patients, Nakamura et al. [11] explicate that the most common postoperative radiological finding after tumour resection through the bifrontal approach was brain oedema found in seven (15.2%) of their patients. Three of these patients died in which two had tumours with a diameter of 4 cm and one a tumour with a diameter of 7 cm.

The results that a pronounced yet increasing oedema translates into higher morbidity and mortality are completely in line with our former experiences. Four patients (50%) who underwent a single-step resection of giant OGMs at our institution between 2000 and 2006 experienced a sudden loss of consciousness after an initially uneventful postoperative course due to an increase of oedema as seen on CT scans. Despite immediate decompressive craniectomy, three patients had an unfavourable outcome, one patient died. This prompted us to alter our strategy for the treatment of giant OGMs in the attempt to improve outcome. Currently, we favour a surgical concept consisting of two steps. As first step, we perform a bifrontal approach and resect about 80% of the anterior part of the tumour via a unilateral transfalcinic approach avoiding ligation of the anterior superior sagittal sinus and leaving behind a small shell-shaped tumour remnant. This proceeding was inspired by an older publication of Kandel and Peresedov [10] pertaining to intracerebral haemorrhages. In order to prevent recurrent bleeding they inserted a silastic balloon into the cavity after stereotactic evacuation of the hematoma and inflated it. Then, they gradually diminished the pressure by removal of a few millilitres of saline solution from the balloon each time over a period of three to four days. With regard to giant OGMs, the leaving of a shell-shaped tumour remnant is not carried out with the intention to prevent secondary bleeding but to minimize the increase of brain oedema as far as possible. One consideration is that after complete removal of a huge space-occupying tumour that was so to speak “chronically” in place for a couple of years the resection cavity that has been created “acutely” may collapse too rapid causing an acute sinking down of the brain and, thus, an increase of the oedema. This acute and rapid descent of the brain is hindered reliably by the tumour remnant. Indeed, it happens that the brain slowly pulsates down the tumour remnant, a feature that also is frequently observed after removal of suprasellar pituitary gland tumours. Another consideration is that the arachnoid is not respected by giant OGMs but disrupted causing the perilesional oedema. Surgical manipulations at the borders of the tumour to the surrounding oedematous brain tissue may significantly increase the perilesional oedema. The one-step resection of the tumours of the patients of our historical group led first by nature to a considerable reduction of the intracranial volume. Despite this fact, four patients suddenly became comatose with clinical signs of cerebral entrapment. Immediate CT scans showed a massive increase of the pre-existing oedema and that the space created by the resection of the tumour was completely used up. Debulking the tumour and leaving behind a shell-shaped tumour remnant as first step in a planned two-step procedure reduces the necessity of surgical manipulations at the tumour borders and, thus, minimizes the increase of brain oedema as far as possible to our point of view. After recovery of the patient and decline of the preexisting oedema, we perform the second step that consists of complete resection of the remainder of the tumour followed by dural and bony reconstruction.

Our clinical results support our rationale. Performing routinely a craniectomy after the fifth patient who deceased, led to a zero mortality in the subsequent patients and a good outcome each time. We have to admit, however, that we cannot provide evidence which of our measures is ultimately responsible for the success. We fully agree with the judgment that previous statements that the anterior third of the superior sagittal sinus can be safely transected without risking serious complications can no longer be supported [11]. Consequently, we did not ligate the sinus in any of our patients. Whether it is now leaving a tumour remnant or the mere effect of craniectomy that translates into good results cannot be decided reliably at this point in time owing to the small number of patients treated this way. In fact, performing a craniectomy, we recently removed a giant OGM completely in the course of the first operation. The patient recovered well and dural reconstruction and reinsertion of the bone flap will follow in a few days. This, however, is also a two-step staged resection of an OGM to our point of view.

One must not ignore the fact, though, that the first operation remains to be major surgery each time. Accordingly, the rate of surgery-related complications encountered in our patients (26.4%) is comparable to the reported frequency in the pertaining literature ranging from 20% up to 36.8% [2, 4, 5, 7, 11, 12, 15]. In addition, since just our method to achieve complete resection of the tumours is different the recurrence rate is similar to other series, too.

## Limitations

The limitation of this study is the relative small number of patients. The rarity of giant OMGs, however, might counteract this disadvantage.

## Conclusion

Our results suggest that a two-step staged resection of giant OGMs with partial resection and craniectomy as first step and complete tumour removal with dural and bony reconstruction thereafter minimizes the increase of postoperative brain oedema as far as possible and translates into lower morbidity and mortality.
